# Comparison of HCV viral load and its genotype distributions in HCV mono- and HIV/HCV co-infected illicit drug users

**DOI:** 10.1186/s12985-017-0797-2

**Published:** 2017-07-11

**Authors:** Marzieh Jamalidoust, Mandana Namayandeh, Mohsen Moghadami, Mazyar Ziyaeyan

**Affiliations:** 1 Department of Virology, Professor Alborzi Clinical Microbiology Research Center, Nemazi Hospital, Shiraz University of Medical Sciences, P. O. Box: 31, Shiraz, 71937-11351 Iran; 2 Department of Virology, Professor Alborzi Clinical Microbiology Research Center, Nemazi hospital, Shiraz University of Medical Sciences, Shiraz, 71937-11351 Iran

**Keywords:** Hepatitis C virus, HIV, HCV genotypes, Illicit drug users, Quantitative PCR assay

## Abstract

**Background:**

Because of shared modes of transmission, patients with hepatitis C virus (HCV) infection are often co-infected with other types of hepatitis viruses and/or HIV. We studied HCV viral load and its genotype patterns among HCV mono- and HCV/HIV co-infected Illicit Drug Users in Fars province-Iran.

**Methods:**

Totally, 580 HCV seropositive IDUs referred to Prof. Alborzi Clinical Microbiology Research Center, Shiraz, Iran, without receiving any anti-HCV treatment, were enrolled. After their HCV infections were reconfirmed by one step rapid diagnostic test, HCV RNA level and HCV genotypes were determined by Taq-man real-time PCR assays. Their HIV serostatus was determined and seropositive patients were excluded from the group.

In addition, 104 HIV/HCV co-infected IDUs referred from Shiraz Behavioral Diseases Consultation Center (SBDC) were assessed for HCV RNA level and HCV genotype patterns, as well.

**Results:**

The overall estimated HIV prevalence was 6.7% (39/580) among HCV seropositive IDUs. Genotype 1, the most prevalent genotype in both groups, was detected in 69% and 49% of co- and mono-infected IDUs, respectively. Median HCV viral load was significantly higher in HIV/HCV co-infected patients, compared with that among HCV mono-infected counterparts.

**Conclusions:**

Given the higher baseline HCV viral load and GT1 attributed to poorer treatments response, HCV treatment must be more considered among HCV/HIV co-infected IDUs, compared to those mono-infected with HCV.

## Background

Hepatitis C Virus (HCV) is the most important cause of liver diseases that may co-occur with Human Immunodeficiency Virus (HIV) [1]. Globally, among 170 million of HCV infected patients, 4-5 million are co-infected with HIV, worldwide [2, 3]. Nowadays, predominant group of HCV infected population are Illicit Drug Users (IDUs) [4]. Syringe sharing and tattooing are the most risk factors in the acquisition of both HCV and HIV infections in IDU sufferers [5].

WHO reports nearly 13 million IDUs exist in the world and 67%, 13% and 8.4% of them are living with HCV, HIV and HCV/HIV co-infection, respectively http://www.who.int/hiv/mediacentre/news/global-aids-update-2016-news/en/. Some studies indicated that 1.2 million permanent addicts and approximately 2.5 million recreational IDUs exist in Iran with HCV and HCV/HIV co-infection frequencies of 45 and 11 %, respectively [6].

Although HCV and HIV can be transmitted by the same route; the rates of their transmission vary in different high risk groups [4]. In IDUs, with small amount of infected blood percutaneous exposure, HCV is approximately 10 times more easily transmitted than HIV and about 30% of HCV infected IDUs have HIV infection, also. Meanwhile, in hemophiliacs, another high risk group with large volume blood transfusion, approximately all HIV infected patients are also co-infected with HCV infection [7].

HCV has appeared as a common cause and a major concern in the treatment of HIV infection after introduction of HAART (High Activity Anti-Retroviral Therapy) therapy [8]. HCV-associated liver dysfunction has become the main cause of morbidity and mortality in IDUs co-infected with HIV [9]. Many studies revealed that HCV viral load, HCV viremia persistency, response to HCV standard therapy and median hepatic fibrosis rate progression in HIV/HCV co-infected patients are significant, compared with those in HCV mono-infected ones [9–11].

Although HCV RNA detection and quantification is the standard method for diagnosis of active HCV infection [3], HCV genotyping is a critical test for determination of treatment duration regime.

In the current study, besides determination of seroprevalence of HIV infection among HCV infected IDUs, HCV viral loads were determined in HCV mono-infected IDUs as well as HIV/HCV co-infected patients. Also, we evaluated HCV GTs in both groups of patients.

## Methods

### Patient’s selection

To be eligible for this study, patients had to be herbal and/or chemical IDUs, be HCV seropositive, age > 15 years with no previous HCV treatment. All included patients have aminotransferase level within the reference range. This study was performed between April 2011 and September 2015, consisting of two main groups a) 580 HCV seropositive IDUs majority from Shiraz university of medical science- Hepatitis clinic and b) 104 HIV/HCV co-infected IDUs referred from Shiraz Behavioral Diseases Consultation Center (SBDC) in Fars Province, southern Iran. All patients referred to Prof. Alborzi Clinical Microbiology Research Center (PACMRC) for more examination. They were interviewed face-to-face and their demographic data and risks were recorded. The liver enzyme level data were checked by reviewing of patients medical records.

Hepatitis clinic, SBDC and PACMRC are institutions affiliated with Shiraz University of medical sciences.

### HCV laboratory diagnostic tests

The presence of specific HCV antibodies in sera samples from infected IDUs referred to PACMRC were reconfirmed with GB anti-HCV V4.0 ELISA kit (Hsinchu science park-Taiwan), according to manufacturer’s guideline.

Five hundred eighty anti-HCV positive specimens as well as 104 anti- HIV/HCV positive samples were further tested with commercially available HCV kits (Genome Diagnostics Pvt. Ltd., Hague, Netherland). In the first step, viral RNA genomes were extracted from 200 μL of HCV mono infected and HCV/HIV co-infected specimens using Invitek kit (Berlin- Germany), according to the guideline described. In the next step, 5 μL of each extracted sample subjected to quantitative HCV RT- PCR test with commercially available HCV quantification kits (Genome Diagnostics Pvt. Ltd., Hague, Netherland), according to the manufacturer’s instructions using 7500 Real-Time PCR system tool (Applied Biosystems, USA), and in the end, HCV genotyping was performed to determine HCV GTs 1-4 among patients who had HCV RNA positive specimens by real-time PCR assay using commercially available kits (Genome Diagnostics Pvt. Ltd., Hague, Netherland) and 7500 Real-Time PCR system tool.

### HIV laboratory diagnostic tests

All HCV seropositive IDUs (with or without HIV infection) referred to PACMRC were screened and/or reconfirmed for HIV by ELISA test (Dia.pro-Milan-Italy). New identified HIV cases in PACMRC were introduced to SBDC as the main HIV consulting center for further hygienic proceeding toward HIV infection.

### Statistical analysis

The data were analyzed using SPSS for Windows systems (Version 16.0, 2007, SPSS Inc., Chicago, IL, United States). We compared Median viral loads in the two groups by Man-Whitney test. We used χ2 test to evaluate HCV GTs patterns.

## Results

As the initial result of this study, it was found that HIV seropositivity rate among HCV infected IDUs was 6.7% (39/580). As mention before, the new identified HIV cases referred to SBDC for further consultation and treatment.

HCV plasma RNA was detected in 79.11% (428/541) and 75% (78/104) of HCV mono- and HCV/HIV co-infected patients, respectively (Table 1), the difference was not statistically significant (*p* ≥ 0.05).

**Table 1:** Demographic and HCV virological status of Iranian HCV infected IDUs with or without HIV infection

Characteristics	HCV seropositive IDUs	HIV/HCV seropositive IDUs
Total number (male/female)	580 (570/10)	104 (93/11)
Mean age	37.8 (SD ± 7.15)	38.3 (SD ± 10.66)
HIV seropositivity rate	6.7%	100%
HCV genome detection rate	79% (428/541)^a^	75% (78/104)
Median viral load among detectable HCV genome^b^	338,880	3549,908^c^

^a^HCV RNA detection was performed after excluded HCV/HIV co-infected patients

^b^Median viral load evaluated among HCV RNA detected sera samples

^c^The viral load unit in our study was copy/ml of serum sample

To investigate our hypothesis that HCV load in HIV/HCV co-infected IDUs is higher than patients who infected only with HCV, the median HCV RNA load was compared in two subjected groups.

**Fig. 1:**
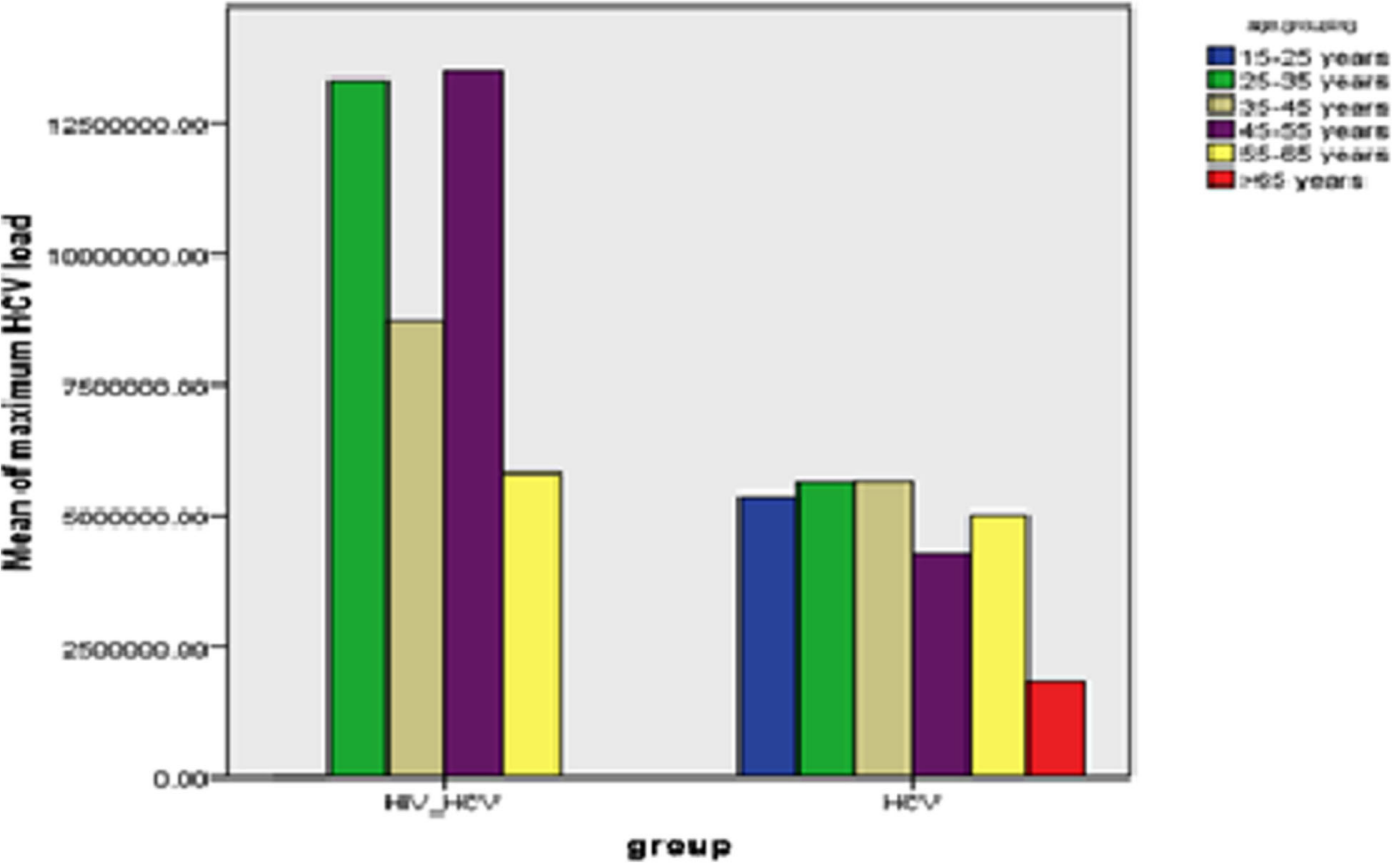
Distributions of HCV mono- and HIV/HCV co-infected among IDUs in different age groups

As shown in Fig. 1 after excluded undetectable HCV genome samples, it was revealed that the median HCV viral load is significantly higher (approximately 10 times) in HCV/HIV co-infected (*n* = 78) IDUs compared with that in HCV mono-infected patients (*n* = 428) (*p* < 0.05).

It’s notable that none of the IDUs had received any HCV treatment before quantification of HCV load and assessment of their HCV GTs.

We also inquire age and sex as potential factors that may be effect on HCV viral load in two subjected groups. Men was predominant in HCV infected IDUs as well as co-infected ones meanwhile 98% and 89% of mono- and co-infected IDUs were male, respectively. The mean age of two groups did not differ significantly.

**Fig. 2:**
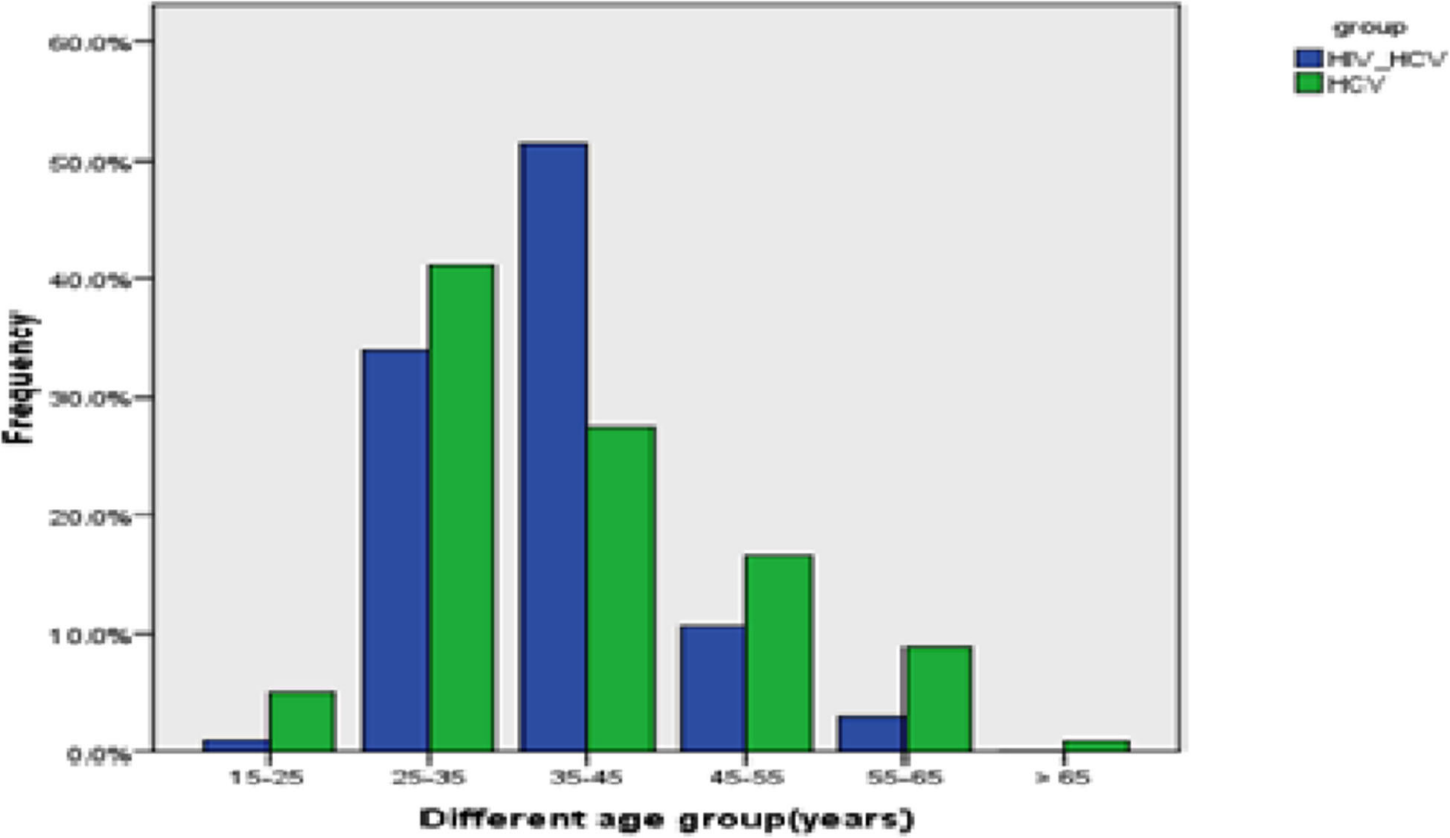
Median of HCV viral loads among HCV mono- and HIV/HCV co-infected IDUs in different age groups

As shown in Fig. 2, 35-45 age group was the most prevalent age range in HIV/HCV affected patients with frequency greater than 50% whereas in HCV mono-infected patients aged 25–35 the infection accounted for about 40%.

We also assessed HCV GTs in the two groups. HCV genotyping was performed on 428/541 HCV mono- and 78/104 HCV/HIV co-infected IDUs, who had detectable HCV RNA. Of them, 348/428 (81.30%) HCV mono-infected and 68/78 (87.17%) HCV/HIV co-infected IDUs were genotyped successfully using HCV genotype real-time kit from Applied Biosystem (Table 2). GT1 was predominant in both of them, but in HCV/HIV co-infected IDUs, GT1 was significantly higher than that in mono-infected counterparts (*p* ≥ 0.5). GT3 was the second most common genotype in both groups but its prevalence was more than approximately 1.5-folds in mono-infected IDUs. There was not seen any mix genotype and GT4 HCV infection in HIV/HCV co-infected patients. Meanwhile 9 HCV mono-infected cases had GT1 of which 7 and 2 were with GT3 and GT2, respectively. The frequency and distribution of HCV GTs among HCV mono and/or co-infected IDUs has been shown in Table 2.

**Table 2:** HCV genotype pattern among Iranian HCV mono- and HCV/HIV co-infected IDUs with detectable HCV RNA

HCV infected IDUs	HCV Genotype distribution					
	Gt1	Gt3	Gt4	Gts 1 + 2/3	No determined Gt	Total
HCV mono-infected	210 (49.06%)	128 (29.9%)	1 (0.23%)	9 (2.10%)	80 (18.69%)	428
HCV/HIV co-infected	54 (69.23%)	14 (17.94%)	0 (0%)	0 (0%)	10 (12.82%)	78
Total	264 (52.17%)	142 (28.06%)	1 (0.19%)	9 (1.77%)	90 (17.78%)	506

According to Table 3, 81/104 (78%) HCV/HIV co- and 295/541 (54%) HCV mono- infected patients had prison life experience (from few months to several months). Tattooing is another risk factor that was seen in 28 and 30% of HCV/HIV co- and HCV mono- infected IDUs, respectively. Multiple sex partners and alcohol drinking were two other risk factors that were evaluated only in co-infected IDUs.

**Table 3:** The most important risk factors and corresponding frequencies in Iranian HCV infected IDUs with or without HIV infection

HCV infected IDUs	Risk factor			
	Imprisonment (%)	Tattoo (%)	Multiple sex partners (%)	Alcohol drinking (%)
HIV/HCV co-infected patients (*n* = 104)	81 (77.9%)	29 (27.8%)	29 (27.9%)	42 (40.3%)
HCV mono-infected patients (*n* = 541)	295 (54.5%)	161 (29.31%)	-	-
Total	384 (56.1%)	185 (27%)	-	-

Totally, as shown in Table 4, nearly 60% of HCV mono-infected and 95% of HCV/HIV co-infected IDUs have experienced more than one risk factor.

**Table 4:** The frequencies of risk factors in HCV mono- and HIV/HCV co-infected IDUs

Illicit drug users^a^	HIV/HCV co-infected patients	HCV mono-infected patients
With none other risk factor	5 (4.8%)	191 (33%)
IDU + 1 another risk factor	46 (44.2%)	301 (52%)
IDU+ 2 other risk factors	26 (25.0%)	39 (6.7%)
IDU+ 3 other risk factors	8 (7.7%)	Not determined
IDU+ 4 other risk factors	13 (12.5%)	Not determined
IDU+ more than 4 risk factors	6 (5.8%)	Not determined
Totally	104 (100%)	531 (100%)

^a^According to the text, risk factors comprise imprisonment, tattooing, having multiple sex partner and alcohols drinking. All of the cases in the study were drug users with or without injection history with up to 4 risk factors

## Discussion

IDUs and hemophiliac patients are the main common HIV/HCV co-infected patients [12–15]. In this study, we determined the HIV seropositive rate among HCV-infected IDUs was 6.7%, consistent with other studies performed in Iran and other countries. In other studies in Shiraz, Honarvar and Davarpanah revealed that the HIV prevalence rates among HCV infected IDUs were 6.43 and 18.3%, that our result was similar to the first one [16, 17] as well as Rahimi-Movaghar and colleagues results which was 8.7% in Tehran [18].

However HCV SVR rate was not evaluated in the current study, but Baseline HCV loads and their GTs are the strongest factors predicting HCV SVR rate. High Mir-122 level, less favorable IL-28 (CT or TT) polymorphism and African-American race are the main host factor leading to poorer SVR [19].

It is notable that in new direct-acting antiviral (DAA) era, the importance of HCV GTs in predicting treatment response has declined significantly.

The present study results were in agreement with other previous studies that showed HCV viral load in HIV/HCV co-infected IDUs was significantly higher than that among HCV mono-infected patients [9, 20, 21]. Mathews et al. showed HCV viral load in HIV/HCV patients was approximately six times greater than that in HCV mono-infected patients [21]. In the present study, the difference was about ten times.

Different studies showed HCV T-cell specific responses decreased significantly in HIV/HCV co-infected patients that were concordant with higher HCV chronic rate in these patients [22, 23].

HCV does not seem to have significant impacts on natural history of HIV infection, however, in post HAART era, according to different large studies; relative liver associated mortality has been increased in HIV/HCV co-infected individuals due to antiretroviral therapy, as a prominent cause. However, AIDS related mortality was decreased dramatically [24].

The results of the current study revealed that HCV GT1 was the most common detected genotype in both mono- and co-infected IDUs with 49 and 69% frequencies, respectively. HCV GT3 was the second most common genotype with 30 and 18% frequencies in mono- and co- infected IDUs, respectively, consistent with our previous study [25].

This study contradicts with Berger et al. study [26] in which no correlation was seen between HIV infection and HCV viral load. The results of the current study revealed a strong correlation between HCV viral load and HIV co-infection as well as GT1 HCV genotype.

Since HCV viral load in HIV/HCV co-infected patients, especially those infected with HCV GT1 is higher than that among mono-infected patients infected with other HCV GTs and more rapid clinical progression in HIV/HCV co-infected patients [11, 27] with delayed sustained virological response (SVR) [15], an efficacious HCV treatment should be considered.

In PHOTON clinical trial, researchers concluded that sofosbuvir plus ribavirin is a more effective regime than standard peg-IFN and ribavirin in the treatment of HIV/HCV co-infected patients and SVR rate was between 84–91%, depending on HCV GTs [28]. However, Torriani et al. has reported that SVR rate was 56% among HIV/HCV co-infected patients 12 week after standard peg-IFN and ribavirin HCV treatment [29].

As shown in Table 3, 53 and 78% of mono- and co-infected IDUs, respectively had variable lengths of prison terms. It can be suggested that these infections with different frequencies are transmitted through the shared needles and drug-preparation equipment in jails.

As indicated in Table 4, only 5% of HIV/HCV co-infected IDUs were with one risk factor (illicit drug using), however, in mono-HCV IDUs this rate was 33%. Ninety one percent (98/104) of HCV mono-infected experienced up to 2 risk factors while 98% (531/541) of HCV/HIV of co-infected IDUs experienced up to 4 risk factors.

In the present study, we did not assess the correlation between CD4+ T-cell count and HCV viral load. As reported in previous studies, the critical point is increasing viral load in patients with impaired cellular immunity, especially HIV positive ones [30, 31]. Further investigations focusing on responses to HCV treatment among both infected groups and other factors such as viral and human genetics, are recommended.

## Conclusion

Iran, one of the countries with a high rate of illicit drug use, has a large number of HCV infected IDUs that may be co-infected with HIV and/or other types of viral hepatitis. We reported a significantly high HCV viral load in HCV/HIV co-infected patients, compared with HCV mono-infected ones. In addition, we showed that in both groups of the patients, GT1 was the dominant genotype followed by GT3. Higher GT1/GT3 ratio and HCV viral load among HCV/HIV co-infected patients suggest more attention to the treatment of such patients.

## Abbreviations

HCV GTs: HCV genotypes
HCV: Hepatitis C Virus
IDUs: Illicit Drug Users
SVR: Sustained Virological Responses
